# Methylglyoxal Induces Inflammation, Metabolic Modulation and Oxidative Stress in Myoblast Cells

**DOI:** 10.3390/toxins14040263

**Published:** 2022-04-07

**Authors:** Sota Todoriki, Yui Hosoda, Tae Yamamoto, Mayu Watanabe, Akiyo Sekimoto, Hiroshi Sato, Takefumi Mori, Mariko Miyazaki, Nobuyuki Takahashi, Emiko Sato

**Affiliations:** 1 Division of Clinical Pharmacology and Therapeutics, Tohoku University Graduate School of Pharmaceutical Sciences, Sendai 980-8578, Japan; soshi.teyo.7295@gmail.com (S.T.); yui.hosoda.s1@dc.tohoku.ac.jp (Y.H.); mayu.watanabe.t3@dc.tohoku.ac.jp (M.W.); take-seki@med.tohoku.ac.jp (A.S.); satou-hrs@jreast.co.jp (H.S.); nobuyuki.takahashi.a8@tohoku.ac.jp (N.T.); 2 Division of Nephrology, Endocrinology, and Vascular Medicine, Tohoku University Graduate School of Medicine, Sendai 980-8574, Japan; tae.yamamoto@med.tohoku.ac.jp (T.Y.); mamiyaza@med.tohoku.ac.jp (M.M.); 3 Division of Nephrology and Endocrinology, Faculty of Medicine, Tohoku Medical and Pharmaceutical University, Sendai 983-8512, Japan; tmori@hosp.tohoku-mpu.ac.jp

**Keywords:** methylglyoxal, sarcopenia, chronic kidney disease, metabolic alteration, myoblast cell

## Abstract

Uremic sarcopenia is a serious clinical problem associated with physical disability and increased morbidity and mortality. Methylglyoxal (MG) is a highly reactive, dicarbonyl uremic toxin that accumulates in the circulatory system in patients with chronic kidney disease (CKD) and is related to the pathology of uremic sarcopenia. The pathophysiology of uremic sarcopenia is multifactorial; however, the details remain unknown. We investigated the mechanisms of MG-induced muscle atrophy using mouse myoblast C2C12 cells, focusing on intracellular metabolism and mitochondrial injury. We found that one of the causative pathological mechanisms of uremic sarcopenia is metabolic flow change to fatty acid synthesis with MG-induced ATP shortage in myoblasts. Evaluation of cell viability revealed that MG showed toxic effects only in myoblast cells, but not in myotube cells. Expression of mRNA or protein analysis revealed that MG induces muscle atrophy, inflammation, fibrosis, and oxidative stress in myoblast cells. Target metabolomics revealed that MG induces metabolic alterations, such as a reduction in tricarboxylic acid cycle metabolites. In addition, MG induces mitochondrial morphological abnormalities in myoblasts. These changes resulted in the reduction of ATP derived from the mitochondria of myoblast cells. Our results indicate that MG is a pathogenic factor in sarcopenia in CKD.

## 1. Introduction

Sarcopenia is a muscle-wasting syndrome characterized by the progressive loss of total body skeletal muscle mass and strength. Uremic sarcopenia, which is chronic kidney disease (CKD)-associated sarcopenia, is a serious clinical problem as it results in physical disability, decreased quality of life, and increased morbidity and mortality [1,2]. The pathophysiology of uremic sarcopenia is multifactorial. The involvement of factors specific to CKD patients, such as insulin resistance, increased inflammatory cytokines, increased oxidative stress, metabolic acidosis, and accumulation of uremic toxins, has been suggested; however, the details remain unknown [3]. Representative protein-bound uremic toxin indoxyl sulfate is markedly accumulated in the plasma of patients with CKD [4] and accumulates in skeletal muscle under CKD conditions [5]. Indoxyl sulfate exerts oxidative and inflammatory activity, triggers aryl hydrocarbon receptor mediated immune responses, and stimulates the progression of CKD [6,7,8,9]. We previously reported that indoxyl sulfate accumulates in muscle tissue and induces metabolic alteration as an antioxidative stress response, resulting in uremic sarcopenia [10]. Recently, Thome T et al. found that several uremic toxins (L-kynurenine, indole-3-acetate, 3-indoleacetonitrile, aminoadipic acid, dimethylarginine, and trimethylamine-*N*-oxide) and numerous potentially novel, unidentified uremic metabolites accumulated in CKD muscle and strongly associated with the degree of mitochondrial impairment [11]. Therefore, it is important to investigate the effects of various uremic toxins on muscle cells, since multiple uremic toxins have the possibility to be implicated in the development and progression of sarcopenia.

Methylglyoxal (MG), a uremic toxin, is a highly reactive dicarbonyl compound that accumulates in circulation in patients with CKD, with or without diabetes [12]. The abnormal accumulation of α-oxoaldehyde metabolites, which is called dicarbonyl stress, is leading to increased modification of protein and DNA with respect to cell and tissue dysfunction in diseases. In addition, MG is a precursor of advanced glycation end products (AGEs) [13,14]. Previous reports suggest that MG impairs the insulin signaling pathways [15] and the interaction between AGEs and the receptor for AGEs (RAGE) obstructs the muscle development program [16]. RAGE signaling is important in skeletal muscle physiology, regulating both the activity of muscle precursors during skeletal myogenesis and the correct timing of muscle regeneration after acute injury [17]. In addition, animal and clinical studies have suggested that AGE levels are increased in circulation and are involved in morphological changes, capillary rarefaction, and mitochondria dysfunction in CKD [18]. In particular, chronic hyperglycemia enhances AGE accumulation in skeletal muscles, and AGEs are correlated with decreased grip strength, leg extension, and slow walking speed [19]. MG plays a role in the development and progression of sarcopenia but the details of the mechanisms, such as whether it induces intracellular metabolic alterations, are not well known. Thus, we hypothesized that MG induces metabolic changes such as indoxyl sulfate with ATP reduction or metabolic flow changes to other pathways, such as lipid metabolism in muscle cells, and contributes to the development of sarcopenia. Since we have previously evaluated the effect of indoxyl sulfate on myocyte metabolism in mouse C2C12 cell line [10], we decided to use the same cell line to determine whether MG induces the same changes in myocyte metabolism as indoxyl sulfate. In the present study, to investigate the mechanisms of MG-induced sarcopenia development and progression, we used a mouse C2C12 cell line to examine the effects of MG on muscle cells, focusing on intracellular metabolism and mitochondrial injury.

## 2. Results

### 2.1. The Effects of MG on Proliferation, Muscle Atrophy, and Oxidative Stress in C2C12 Cells

**Figure 1:**
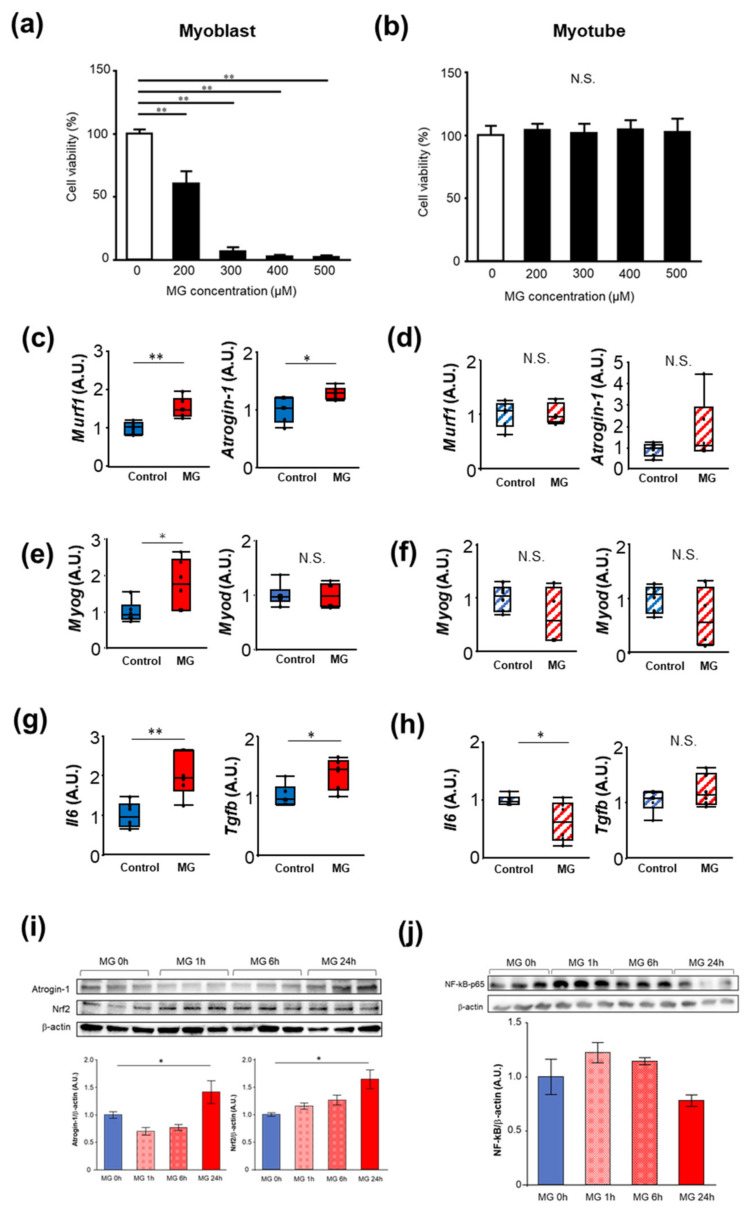
Methylglyoxal (MG) inhibits cell proliferation in C2C12 myoblast cells. C2C12 myoblast cells (**a**) and myotube cells (**b**) were exposed to MG at the indicated concentrations for 24 h; this was followed by MTT (3-(4,5-dimethylthiazol-2-yl)-2,5-diphenyltetrazolium bromide, yellow tetrazole) assay (*n* = 8). The mRNA levels in the C2C12 myoblast cells (**c**,**e**,**g**) and the C2C12 myoblast cells (**d**,**f**,**h**) (*n* = 6) treated with or without MG for 24 h. The protein levels in the C2C12 myoblast cells treated with or without MG for 1, 6, 24 h (**i**,**j**). A.U.; arbitrary unit. N.S.; not significant, * *p* < 0.05, ** *p* < 0.01, difference with control by Student’s *t*-test.

To examine the mechanism underlying MG in the skeletal muscle in uremic sarcopenia, the mouse myoblast cell line C2C12 and differentiated myotubes were used. We examined the toxic effects of MG on skeletal muscles using C2C12 cells. Since circulating MG concentration increases to the μM order during renal failure [4], these concentrations were selected for the present study. The cell viability of the C2C12 myoblast cells significantly decreased under MG-treated conditions than under control conditions in a concentration-dependent manner (Figure 1a); however, the viability of the C2C12 myotube cells was not significantly different (Figure 1b). We also examined the effect of MG on the expression of muscle atrophy-associated genes in C2C12 myoblast cells and C2C12 myotube cells. MG significantly increased the mRNA levels of muscle RING finger 1 (Murf1) and muscle atrophy F-box (Atrogin-1), a muscle-specific E3 ubiquitin ligase in C2C12 myoblast cells; however, the levels of these mRNAs were not significantly different in the C2C12 myotube cells (Figure 1c,d). Next, we examined the effects of MG on myogenic differentiation. MG significantly increased the mRNA levels of myogenin (*Myog*) in the C2C12 myoblasts. In contrast, MG did not affect the mRNA level of myoblast determination protein 1 (*Myod*) in the C2C12 myoblast cells. The mRNA levels of *Myog* and *Myod* were not significantly different in the C2C12 myotube cells after MG exposure (Figure 1e,f). Furthermore, we examined the mRNA levels of the inflammation marker, Il6, and fibrosis marker, Tgfb, in C2C12 myoblast cells and C2C12 myotube cells. MG significantly increased the mRNA levels of Il6 and Tgfb in the C2C12 myoblast cells, although the mRNA level of Tgfb was not significantly different in the C2C12 myotube cells (Figure 1g,h). MG significantly decreased the mRNA level of Il6 in the C2C12 myotube cells. Furthermore, we examined the protein levels of atrogin-1, which is involved in muscle atrophy, and transcription factor nuclear factor kappa-light-chain-enhancer of activated B cells (NF-kB) involved in its expression, and nuclear factor (erythroid-2-related factor)-2 (Nrf2), which is the main transcriptional activator in response to oxidative stress [20] (Figure 1i,j). MG significantly increased protein level of atrogin-1 for 24 h stimulation, and NF-kB level was increased by MG stimulation from 1 to 6 h. In addition, MG increased the protein level of Nrf2 in the C2C12 myoblast cells to a maximum level 24 h after exposure (Figure 1i).

**Figure 2:**
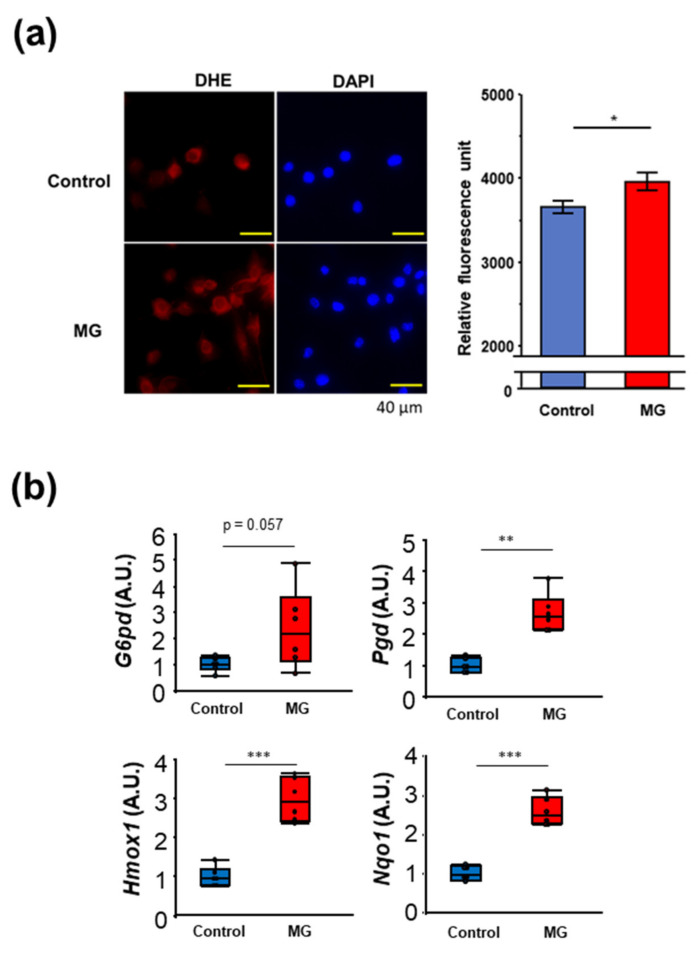
Methylglyoxal (MG) induces oxidative stress. (**a**) Relative fluorescence unit of dihydroethidium (excitation/emission: 518/606 nm) in C2C12 myoblast and representative images with and without MG. (**b**) Relative expression of nuclear factor erythroid 2-related factor 2 direct target genes, glucose-6-phosphate dehydrogenase (*G6pd*), phosphogluconate dehydrogenase (*Pgd*), hemeoxygenase-1 (*Hmox-1*), and NAD(P)H quinone dehydrogenase 1 (*Nqo1*) in the C2C12 myoblast cells. A.U.; arbitrary unit. * *p* < 0.05, ** *p* < 0.01, *** *p* < 0.001, difference with control by Student’s *t*-test.

Next, we examined the toxic effects of MG on myoblast cells. Because MG is a highly reactive dicarbonyl compound that is known to trigger oxidative stress, and MG induced Nrf2 expression, we examined the oxidative stress induced by MG in the C2C12 myoblast cells using dihydroethidium (DHE). Treatment with MG resulted in increased DHE fluorescence compared to the control (Figure 2a). We then examined the expression of Nrf2 direct targets, such as glucose-6-phosphate dehydrogenase (G6pd), phosphogluconate dehydrogenase (Pgd), hemeoxygenase-1 (Hmox-1), and NAD(P)H quinone dehydrogenase 1(Nqo1) in the C2C12 myoblast cells. Consistent with the time course of Nrf2 protein levels, the expression of G6pd, Pgd, Hmox-1, and Nqo-1 were significantly increased in MG-treated C2C12 myoblast cells (Figure 2b). These results suggest that MG suppresses proliferation and induces muscle atrophy, inflammation, differentiation, and oxidative stress in C2C12 myoblast cells.

### 2.2. MG Reduced Levels of Tricarboxylic Acid (TCA) Cycle Intermediates and Induced ATP Shortage in C2C12 Myoblast Cells

**Figure 3:**
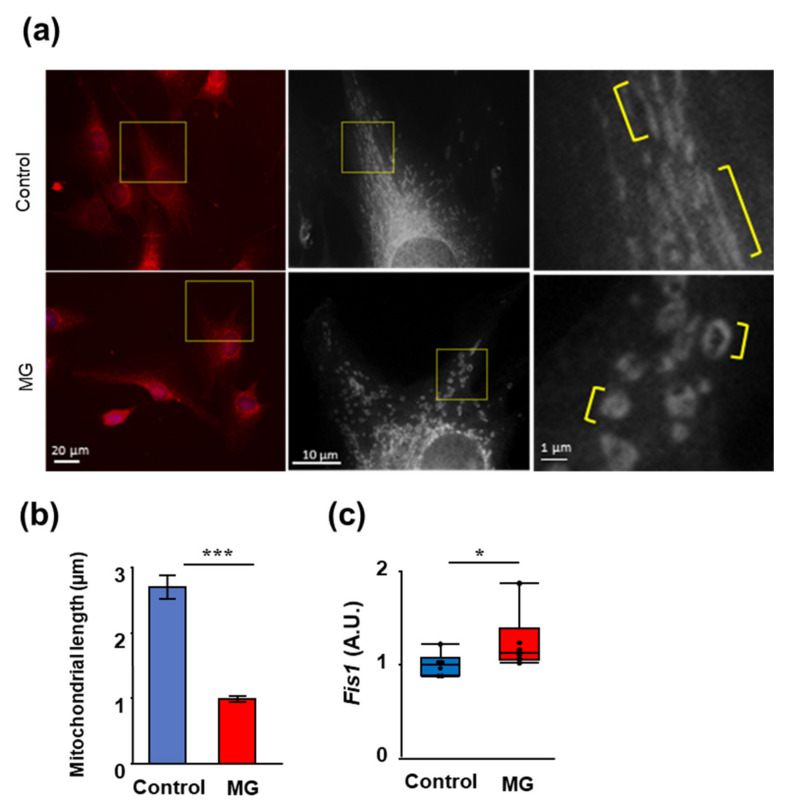
Methylglyoxal (MG) induces mitochondrial abnormality. (**a**) Representative images of the mitochondria in C2C12 myoblast cells stained with 200 nM Mitotracker Red for 30 min and exposed or unexposed to 200 mM MG. The yellow square box is shown enlarged (right and middle). The yellow line indicates the mitochondrial length (left). (**b**) Quantitative analysis (mean ± standard deviation; *n* = 20) of mitochondrial length. Data are the mean ± standard error, *** *p* < 0.001, the difference compared with control as analyzed by the Student’s *t*-test. (**c**) Relative mRNA levels of fission protein 1 (*Fis1*) normalized with *Gapdh*. Data are boxplots, * *p* < 0.05, the difference compared with control, as analyzed using Kruskal–Wallis test; *n* = 6.

Next, we evaluated mitochondrial morphology to examine mitochondrial abnormalities induced by MG, because mitochondrial dysfunction is a key mechanism for atrophy progression in skeletal muscle. Mitochondrial morphology changed when exposed to MG, compared to the control. Mitochondrial length in the C2C12 myoblasts was significantly shortened by MG treatment (Figure 3).

**Figure 4:**
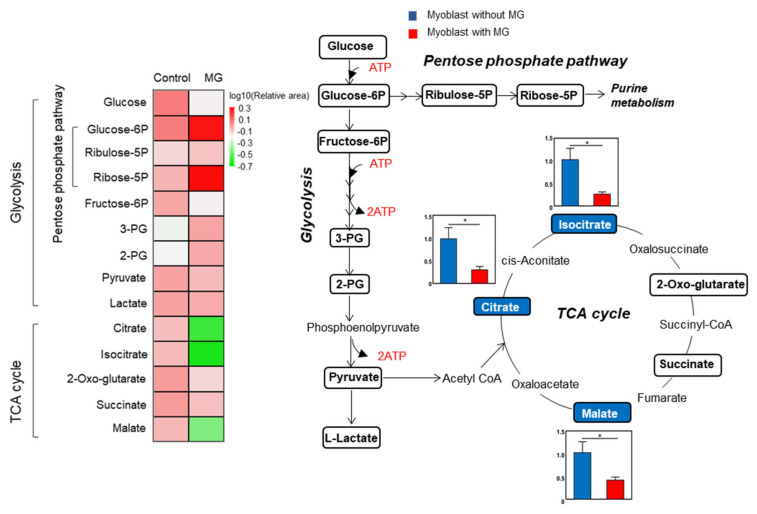
Metabolic response of C2C12 myoblast cells upon exposure to methylglyoxal (MG). Relative changes in metabolite levels in glycolysis and tricarboxylic acid cycle comparing non-treated (blue) and 200 μM MG-treated (24 h, red) C2C12 myoblast cell extracts are shown. Data are the mean ± standard error of the mean, * *p* < 0.05, difference analyzed using the Student’s *t*-test; *n* = 6.

Furthermore, we investigated the effects of MG on intracellular glucose metabolic changes in skeletal muscle using C2C12 myoblast cells. Metabolic changes in C2C12 myoblast cells treated with or without MG were analyzed using targeted metabolomics with gas chromatography-mass spectrometry (GC-MS). Figure 4 shows the metabolites in glycolysis and the TCA cycle and a heatmap of these metabolites. The levels of metabolites in glycolysis were not significantly different between the control and MG exposure groups. In contrast, the levels of the TCA cycle intermediates, citrate, isocitrate, and malate were significantly decreased in C2C12 myoblast cells exposed to MG for 24 h compared to the control group, suggesting that the TCA cycle was suppressed, but glycolysis was maintained.

**Figure 5:**
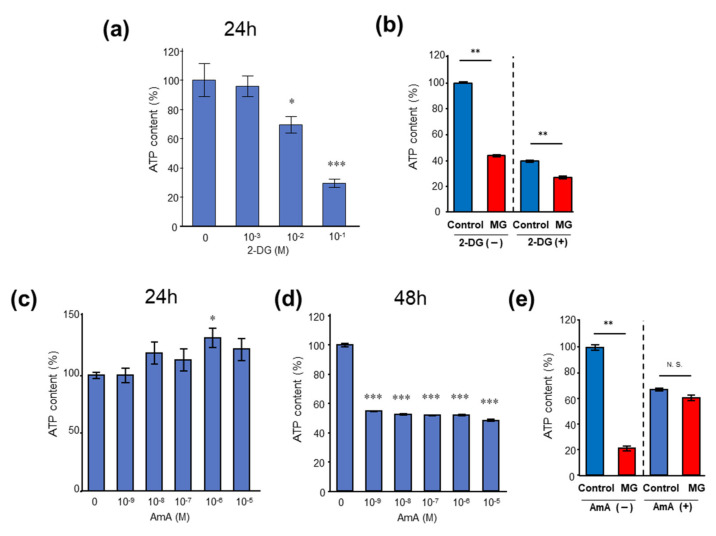
ATP contents in C2C12 myoblast cells. (**a**) Effects of 2-deoxyglucosone (2-DG) on ATP concentrations of C2C12 myoblast cells. (**b**) ATP content in the C2C12 myoblast cells exposed to 200 μM MG for 24 h with or without the glycolysis inhibitor, 100 mM 2-DG. (**c**,**d**) Time-dependent and dose-dependent effects of antimycin A (AmA) on C2C12 myoblast cells. (**e**) ATP content in the C2C12 myoblast cells with or without mitochondrial inhibitor, 10 μM AmA, treatment for 48 h in the presence of 200 μM MG for 24 h. Data are the mean ± standard error of the mean, * *p* < 0.05, difference using the Student’s *t*-test; *n* = 6. N.S.; not significant, * *p* < 0.05, ** *p* < 0.01, *** *p*< 0.001, difference with 0 by Dunnett’s test (**a**,**c**,**d**) or difference with Kruskal–Wallis test (**b**,**e**).

Thus, we examined the ATP content in C2C12 myoblast cells to determine whether MG affects only the TCA cycle. To assess the toxicity of MG to mitochondria in myoblast cells, C2C12 myoblast cells were cultured in the presence of 1 mM–100 mM 2-deoxyglucose (2-DG), which is a glycolytic inhibitor. In the presence of 10 mM and 100 mM 2-DG, ATP levels were significantly reduced (Figure 5a). In the presence of 100 mM 2-DG, ATP levels were reduced to 40% of the control condition, and ATP levels were further reduced with MG (Figure 5b). Next, to examine the effect of MG on glycolysis in C2C12 myoblast cells, the cells were treated with 0.001–10 μM antimycin A (AmA), which is a mitochondrial electron transport blocker. No significant decrease was observed in the 24 h co-culture at any concentration of AmA (Figure 5c). Co-culture with AmA for 48 h significantly decreased ATP content at all concentrations compared with the control condition (Figure 5d). In the presence of 10 μM AmA, ATP levels were reduced to 65% of the control condition, and no significant difference was observed with MG (Figure 5e). These results suggest that MG induces mitochondrial damage and ATP shortage, and affects only the TCA cycle, but not glycolysis in C2C12 myoblast cells.

**Figure 6:**
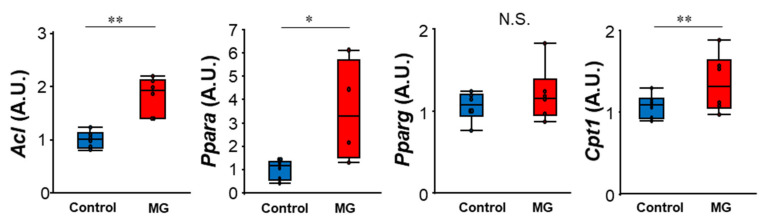
mRNA levels in C2C12 myoblast cells. ATP citrate lyase (*Acl*), peroxisome proliferator-activated receptor-γ (*Pparg*), peroxisome proliferator-activated receptor-α (*Ppara*), and carnitine palmitoyltransferase 1 (*Cpt1*)- expression normalized with that of *Hprt*. N.S.; not significant, * *p* < 0.05, ** *p* < 0.01, difference analyzed using the Student’s *t*-test; *n* = 6.

Next, we investigated why the levels of citrate, isocitrate, and malate were reduced by MG. Citrate is a major source of cytosolic acetyl CoA required for the biosynthesis of fatty acids and cholesterol. In the cytosol, citrate is cleaved by ATP-citrate lyase (*Acl*) into acetyl CoA and oxaloacetic acid, and acetyl CoA is carboxylated and imported into the synthesis of fatty acids/cholesterol. Peroxisome proliferator-activated receptor alpha (*Ppara*) is a transcription factor and a major regulatory factor of lipid metabolism. Peroxisome proliferator-activated receptor gamma (*Pparg*) is a transcription factor that also regulates fatty acid storage and glucose metabolism. Carnitine palmitoyltransferase 1 (*Cpt1*) acts as a rate-limiting enzyme for the transport of long-chain fatty acids into the mitochondria and subsequent β-oxidation, playing a central role in the regulation of energy metabolism. The mRNA levels of *Acl*, *Ppara*, and *Cpt1,* but not *Pparg*, were significantly increased in C2C12 myoblast cells exposed to MG (Figure 6). These results suggest that, after MG exposure, glucose metabolic flow is changed to the glycolysis-citrate-fatty acid synthesis pathway from the glycolysis-TCA cycle-electron transport chain.

## 3. Discussion

**Figure 7:**
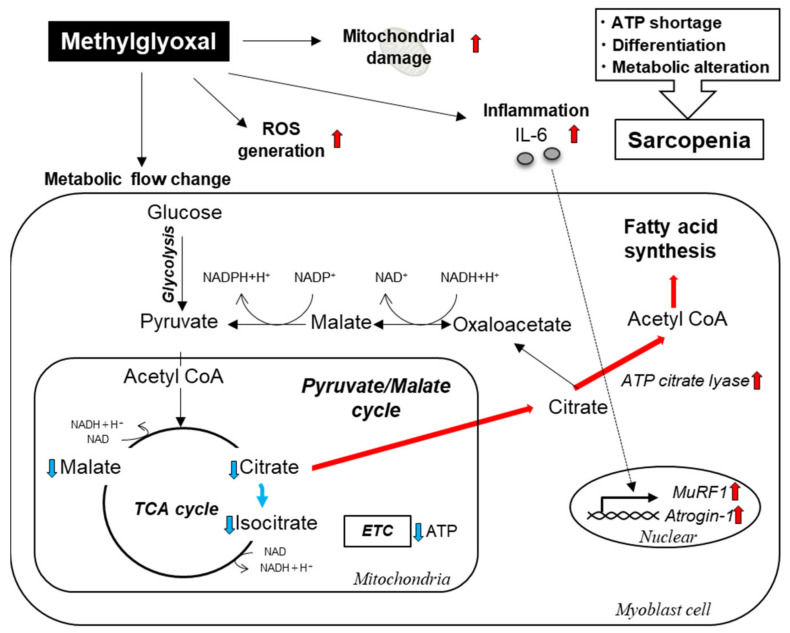
Schematic illustration of the effects of methylglyoxal (MG) in myoblast cells. MG induces metabolic flow change (such as switching to the glucose-citrate-fatty acid synthesis pathway) and mitochondrial damage, reactive oxygen species generation, interleukin-6 increase, and activation of the muscle atrophy pathway.

In the present study, we found that (1) MG reduced viability of the C2C12 myoblast cells but not of the myotube cells; (2) MG induced the expression of genes related to muscle atrophy, inflammation, fibrosis, and oxidative stress; and (3) MG induced alteration of intracellular metabolism with mitochondrial morphological changes and ATP content reduction in myoblast cells. A novel finding of this study is that MG affects only myoblast cells via the activation of the muscle atrophy pathway (MuRF1 and Atrogin-1), where it causes a metabolic change from the TCA cycle to fatty acid synthesis, resulting in a reduction of ATP levels. These observations suggest that MG may be related to the development and progression of CKD-associated sarcopenia through the reduction of myoblast cell population via intracellular metabolic alterations (Figure 7). Although the pathogenesis of sarcopenia is multifactorial, disruption of the balance between the degradation (catabolism) and synthesis (anabolism) of skeletal muscle proteins is deeply involved in the development and progression of sarcopenia [3]. In uremic sarcopenia, various factors, such as inflammation and metabolic acidosis, activate the ATP-dependent ubiquitin-proteasome system (UPS) [2,3]. The ubiquitin ligase (E3) enzymes of the UPS, muscle RING-finger protein 1 (MuRF1), and Atrogin-1, which play important roles in the strict selection of proteins for degradation, are involved in sarcopenia [21]. The loss of muscle mass, or muscle atrophy, is a complicated process that occurs as a consequence of a variety of stressors, such as inflammation, oxidative stress, and abnormal metabolism. Myostatin, a subfamily of transforming growth factor-beta, binds to activin IIB receptors on myocytes and activates Smad2/3, transcription factors that bind to DNA and are involved in the regulation of *Murf1* and *Atrogin-1* expression [22]. Tumor necrosis factor-α, an inflammatory cytokine, increases the expression of myostatin via the nuclear factor kappa-light-chain-enhancer of activated B cells-dependent pathway, and myostatin induces the production of interleukin (IL)-6 via p38 mitogen-activated protein kinase (MAPK) and MAPK kinase 1 [23]. IL-6 activates various signaling pathways including the muscle atrophy pathway [24], and a longitudinal study in elderly human subjects has shown that high serum IL-6 levels increase the risk of muscle strength loss [25]. Thus, our results indicate that MG induces inflammation and activates ATP-dependent UPS in myoblast cells.

In our study, mRNA levels of *Il6* increased in myoblast cells and decreased in myotube cells under MG exposure. IL-6 has been recognized as a myokine that plays an essential role in the skeletal muscle [26,27]. A previous animal study reported that the overexpression of circulating IL-6 promotes the production and accumulation of free radicals in the diaphragm muscle by regulating redox-associated molecular circuits and impinging the Nrf2 mediated antioxidant response [28]. IL-6 affects myoblast proliferation; however, its effect on differentiation is still being investigated [26]. Recently, a study revealed that low IL-6 concentrations induce proliferation, whereas high IL-6 concentrations induce differentiation in both C2C12 mouse myoblasts and primary human myoblasts [27]. Consistent with previous reports, the expression of *Myog* increased in myoblast cells in which *Il6* expression increased under MG exposure. In muscle injury and wasting skeletal muscle disease, muscle satellite cells undergo activation, proliferation, and eventual differentiation [29,30,31]. Several cytokines are considered to be involved in satellite cell activation, including the IL-6 superfamily. MyoG is eventually expressed when myoblast cells are completely committed to differentiation, and MyoG expression is thus a late phenomenon [32]. In other words, our study suggested that MG upregulates *Il6* expression, inhibits cell proliferation, and promotes myoblast cell differentiation into myotubes. On the other hand, *Il6* expression is downregulated by MG in myotubes, indicating that it may suppress the differentiation of myotubes to myofiber; however, the effects of IL-6 on differentiation are not well understood.

MG increased reactive oxygen species (ROS) and Nrf2 protein levels and the expression of downstream genes in myoblast cells in the present study. In our previous in vitro study, it was shown that methyl radicals, oxy-carbon centered radicals, and hydroxyl radicals are generated via non-enzymatic reactions between MG and hydrogen peroxide [33]. In the pathological process of radical generation, methyl radicals are readily oxidized by dissolved oxygen to form peroxide radicals, which are involved in the peroxidation reaction. Nrf2 is a well-known transcription factor that regulates intracellular antioxidants and detoxification enzymes [20,34]. Recently, it was reported that the Nrf2/Keap1 pathway is important in the MG detoxification mechanism against MG-induced carbonyl stress in neurons, and Nrf2 activators contribute to the accumulation of carbonyl stress mediators and the suppression of toxic expression [35]. This suggests that MG generates free radicals and increases ROS levels, and Nrf2 is activated to prevent MG-induced cytotoxicity and protein modifications in myoblast cells. Mitochondria play very important roles in skeletal muscle, such as energy supply, ROS production, and calcium homeostasis [36]. In the present study, MG induced mitochondrial morphological abnormalities in myoblast cells. Muscle biopsies of patients with CKD-associated sarcopenia showed the reduction of mitochondrial proteins [37], a decreased mitochondrial volume density, and decreased mitochondrial biogenesis/mass indices [38]. The association between mitochondrial dysfunction and sarcopenia has previously been reported in both patient and animal models of CKD [37,39,40]. In addition, when skeletal muscle is damaged, myoblast cells differentiate into myotube cells and fuse to the damaged area for repair to maintain skeletal muscle [41]. During myoblast differentiation, ATP levels gradually increase and reach 200% of the baseline levels. Thus, ATP plays an important role in myoblast differentiation into myotubes. Our metabolomics data of intramuscular cells showed that MG reduced the levels of citrate, isocitrate, and malate, which are TCA substrates, and caused ATP shortage. This means, after MG exposure, glucose metabolic flow changed from the glycolysis-TCA cycle-electron transport chain to the glycolysis-citrate-fatty acid synthesis pathway in myoblast cells. Further experiments with glycolytic or mitochondrial inhibitors showed that MG significantly reduced mitochondrial-derived ATP production in the present study. In myopathies, including age-related sarcopenia, ectopic lipogenesis commonly occurs [42]. In the pathogenesis of adipose degradation, stem cells present in muscle tissue have been reported to function as a source of adipocytes [43]. Adipogenesis can be induced by several types of cells, including satellite cells isolated from the skeletal muscles [44,45]. C2C12 myoblast cells, which were used in the present study, have been reported to be influenced by several factors such as cytokines [46,47]. Our data did not detect lipogenesis in myoblast cells following MG exposure; however, metabolic flow occurred through the glucose-citrate-fatty acid synthesis pathway after MG exposure in myoblast cells. Thus, MG can induce ectopic lipogenesis in myoblasts. To validate this hypothesis, further studies on lipidomics are required. Thus, our work suggests that MG may be related to the development and progression of CKD-associated sarcopenia through the reduction of myoblast cells via intracellular metabolic alterations. Therefore, in combination with our previous findings on the effects of indoxyl sulfate [10], uremic-toxin-induced metabolic changes in myocytes may play a key role in CKD-associated sarcopenia. Our results suggested that targeting these alterations may be helpful for the prophylaxis or therapy of uremic sarcopenia. In the present study, we choose C2C12 cell line, because we have previously studied uremic toxin-induced metabolic alterations using C2C12 cell line [10]. There are more than 130 uremic toxins have been reported until today, and we speculate that each of them has different effects. Therefore, we used C2C12 cell line to investigate the effects different uremic toxins. The results of this study were obtained using cells of animal origin and have research limitations. To validate the findings obtained in the present study, further studies are required to clarify whether similar alterations are observed in muscle tissues of CKD animal models and patients with CKD.

## 4. Conclusions

MG-induced intramuscular metabolic alterations such as metabolic flow change to fatty acid synthesis and the reduction of TCA cycle substrates yielding ATP shortage are pathogenic factors for the development of CKD-associated sarcopenia.

## 5. Materials and Methods

### 5.1. Cell Culture

The mouse C2C12 myoblast cell line was obtained from the American Type Culture Collection (Manassas, VA, USA) and grown in Dulbecco’s Modified Eagle Medium (DMEM) (Thermo Fisher Scientific, Waltham, MA, USA) containing 10% fetal bovine serum, 100 IU/mL penicillin, 100 IU/mL streptomycin, and 1 mM L-glutamine in a humidified incubator at 37 °C with 5% CO_2_ in the air. Confluent C2C12 myoblasts were differentiated into myotubes by incubation with DMEM containing 2% horse serum for 4–5 days.

### 5.2. Cell Proliferation Asay

To assess the effects of MG on skeletal muscle cell growth and viability, the 3-(4,5-dimethylthiazol-2-yl)-2,5-diphenyltetrazolium bromide assay (V13154; Thermo Fisher Scientific, Waltham, MA, USA) was performed according to the manufacturer’s protocol. Cells seeded in 96-well plates were treated with different concentrations of MG for 24 or 48 h.

### 5.3. Polymerase Chain Reaction (PCR) Analysis

Total RNA was extracted using an RNeasy Mini kit (Qiagen, Hilden, Germany) according to the recommended protocol. Extracted RNA was reverse transcribed to complementary DNA (cDNA) using the iScript Advanced cDNA Synthesis Kit for RT-qPCR (Bio-Rad Laboratories, Hercules, CA, USA) according to the recommended protocol. PCR was performed in a total volume of 1.5 μL containing aliquots of cDNA, 0.45 μL of 10 μM of each primer, and SsoAdvanced Universal SYBR Green Supermix (Bio-Rad Laboratories, Hercules, CA, USA). After heating at 95 °C for 3 min, denaturation, annealing, and elongation were carried out at 95 °C for 3 min, 95 °C for 5 s, and 60 °C for 15 s, respectively. Reactions were repeated for 39 cycles. Expression of hypoxanthine phosphoribosyltransferase (Hprt) mRNA was used as an internal control. The genes of interest (Hprt, Gapdh, Trim63; Murf1, Fbxo32; Atrogin 1, Il6, Tnfa, Tgfb, Hmox1, and Nqo1) were obtained from Takara (Kusatsu, Japan), and their set IDs were Hprt: MA031262, Trim63: MA056880, Fbxo32: MA155273, Il6: MA15227, Tnf: MA165780, Tgfb: MA148599, Hmox1: MA141757, Nqo1: MA121914, Gapdh: MA050371, Myod1: MA128901, and Myog: MA127738. The primer sequences for Fis1, Pparg, and Acl were as follows: Fis1 Forward: CCGGCTCAAGGAATATGAAA and Reverse: CCATGCCTACCAGTCCATCT, Pparg forward: ATGGAGCCTAAGTTTGAGTT and reverse: CAGCAGGTTGTCTTGGATGT; Acl forward, TGGATGCCACAGCTGACTAC; and reverse, GGTTCAGCAAGGTCAGCTTC.

### 5.4. Mitochondrial Morphology Analysis

C2C12 cells were incubated with 100 nM MitoTracker Red CMXRos (Invitrogen, Waltham, MA, USA) for 30 min. An all-in-one fluorescence microscope (BZ-X800; Keyence, Osaka, Japan) was used to obtain images.

### 5.5. ROS Detection

ROS levels were determined using the fluorescent dye DHE (Thermo Fisher Scientific, D11347, Waltham, MA, USA). C2C12 cells were incubated with 10 μM DHE for 30 min. An all-in-one fluorescence microscope (BZ-X800; Keyence, Osaka, Japan) was used to obtain images. Quantitative analysis was performed using a fluorescence plate reader (SpectraMax iD5, Molecular Devices, San Jose, CA, USA), and data are presented as fluorescence intensity.

### 5.6. Biochemical Measurement

The cellular ATP content of C2C12 cells was measured using a luminometric ATP assay kit (TOYO B-Net Inc., Tokyo, Japan) according to the manufacturer’s protocols. Briefly, cells were treated with MG for 24 h before 100 μL/well of the ATP reaction mixture was added to the sample and mixed gently. Luminescence intensity was detected at 23 °C using a SpectraMax L microplate reader (Molecular Devices, San Jose, CA, USA). When measuring mitochondrial-derived ATP, 100 mM 2-DG was added to the medium with MG for 24 h. Treatment with 100 mM 2-DG significantly suppressed ATP production in the C2C12 myoblasts. When measuring glycolysis-derived ATP, 10 μM AmA was added and incubated for 24 h. Following 24 h of incubation, MG was added and incubated for a further 24 h, and ATP was measured. Treatment with 10 μM AmA for 48 h, but not for 24 h, significantly suppressed ATP production in the C2C12 myoblasts.

### 5.7. Western Blotting

Western blotting was performed according to our previously reported methods [10]. Proteins were extracted using 1X radioimmunoprecipitation assay buffer (Cell Signaling Technology, Danvers, MA, USA) containing a protease inhibitor (Roche Diagnostics K. K., Tokyo, Japan), phosphatase inhibitor cocktail (Sigma Aldrich, St. Louis, MO, USA), and 1 mM phenylmethylsulfonyl fluoride (Thermo Scientific, Waltham, MA, USA). Ten μM MG132 (Sigma Aldrich, St. Louis, MO, USA) was added into the extraction buffer to protect it from degradation. Quick Start protein assay (Bio-Rad Laboratories, Hercules, CA, USA) was used for protein concentration determination. Fifteen μg of protein was used for each sodium dodecyl sulfate polyacrylamide gel electrophoresis run. The anykD Mini-Pro TEAN Precast Gel (Bio-Rad Laboratories, Hercules, CA, USA) was used for each analysis. The protein extracts were transferred onto a polyvinylidene difluoride membrane. After blocking for 1 h, the membrane was incubated with primary antibodies (anti-NRF2, 1:200, #14596, Cell Signaling or anti-atrogin-1, 1:1000, MK6170ECM Biosciences) overnight at 4 °C. After washing, the membrane was incubated with secondary antibodies (anti-rat IgG, sc-2032, 1:5000, Santa Cruz or anti-rabbit Ig, MK6170, 1:5000, ECM Biosciences) for 1 h at 25 °C. The expression of β-actin (1:5000, sc-47778, Santa Cruz Biotechnology, Dallas, TX, USA) was used as an internal control.

### 5.8. Sample Preparation for GC-MS Measurement

To measure the levels of metabolites in C2C12 myoblasts and myotube cells, an aliquot (750 μL) of methanol containing 1 mg/mL citrate-d_4_ was added to 1 × 10^5^ cells to extract cellular metabolites, and the resultant solution was moved to a 1.5-mL tube. The samples were vortexed for 5 min and then frozen in liquid nitrogen for 1 min. They were then dissolved at room temperature for 5 min and sonicated for 5 min. The above process was repeated a total of three times and samples were then centrifuged at 20,400× *g* at 4 °C for 15 min. The supernatant was collected in a 1.5-mL tube, and the remaining cell pellet was lysed with 0.25 mL of MilliQ water, vortexed, and placed on ice for 10 min. The cells were then centrifuged under the same conditions as before, and the supernatant was collected in a 1.5-mL tube. The remaining cell pellet was stored frozen at −80 °C for protein quantification. An amount of 10 μL of 0.5 mg/mL isopropylmalic acid was added to a 1.5-mL tube containing the supernatant and vortexed. Then, 500 μL was dispensed from the tube, and the tubes were decompressed and dried in an evaporator. Next, 80 μL of 20 mg/mL methoxyamine hydrochloride (136-05933; FUJIFILM Wako Pure Chemical Corp., Osaka, Japan) pyridine solution was added to the sample, which was then sonicated for 20 min and shaken at 1200 rpm for 90 min at 30 °C. Then, 40 μL of N-methyl-N-trimethylsilyl-trifluoroacetamide (1022-11061; GL Science, Tokyo, Japan) was added, and the solution was shaken for 30 min at 1200 rpm at 37 °C. After shaking, the samples were centrifuged at 16,000× *g* for 3 min at 4 °C and the supernatant was used as a sample for GC-MS measurements.

### 5.9. GC-MS Measurement

This measurement was based on our previously reported methods [48]. GC-MS analysis was performed using a GC-MS QP2010 Ultra (Shimadzu Corp., Kyoto, Japan) with a fused silica capillary column (BPX-5; 30 m × 0.25 mm inner diameter, film thickness: 0.25 μm; Shimadzu Corporation, Kyoto, Japan) and a front inlet temperature of 250 °C and helium gas flow rate through a column of 39.0 cm/s. The column temperature was held at 60 °C for 2 min, then raised by 15 °C/min to 330 °C and maintained for 3 min. The interface and ion-source temperatures were 280 °C and 200 °C, respectively. To perform a semi-quantitative assessment, the peak height of each quantified ion was calculated and normalized using the citrate-d_4_ and 2-isopropylmalate peak heights and protein concentrations. The retention times and selected reaction monitoring conditions of the derivatized metabolites are summarized in Table S1.

### 5.10. Statistical Analysis

JMP Pro software version 16.0.0 (SAS Institute Inc., Cary, NC, USA) was used for statistical analysis. All values are expressed as box plots unless otherwise stated. Differences were considered statistically significant at *p* < 0.05. Statistical significance was evaluated using the Student’s *t*-test or the Tukey-Kramer test with the analysis of variance for normally distributed variables and Kruskal-Wallis test for non-normal distributions.

## Data Availability

Not applicable.

## Supplementary Materials

The following supporting information can be downloaded at: https://www.mdpi.com/article/10.3390/toxins14040263/s1, Table S1: Measurement conditions for GC-MS measurements.
