# Supplementary material for: Methylglyoxal Induces Inflammation, Metabolic Modulation and Oxidative Stress in Myoblast Cells
Source: Toxins (Basel). 2022 Apr 7;14(4):263. doi: 10.3390/toxins14040263 (PMC9030564; doi:10.3390/toxins14040263)
Supplement: Supplementary file 1 [file toxins-14-00263-s001.zip › toxins-1618492-supplementary.pdf]

toxins

MDPI

Methylglyoxal induces inflammation, metabolic Modulation, and oxidative stress in myoblast cells

Sota Todoriki, Yui Hosoda, Tae Yamamoto, Mayu Watanabe, Akiyo Sekimoto, Hiroshi Sato, Takefumi Mori, Mariko Miyazaki, Nobuyuki Takahashi and Emiko Sato

Table S1. Measurement conditions for GC-MS measurements.

Analyte	Retention time (min)	Ion (MS)
Glucose-meto-5TMS	13.429	319.10, 205.1
Glucose 6-phosphate-meto-6TMS	16.041	471.2, 387.2
Ribulose 5-phosphate-meto-5TMS	14.762	357.1, 299.1
Ribose 5-phosphate-meto-5TMS	14.787	459.2, 315.1
Fructose 6-phosphate-meto-6TMS	15.953	459.2, 357.1
3-Phosphoglycerate-4TMS	12.863	459.1, 387.2
2-Phosphoglycerate-4TMS	12.657	459.1, 387.2
Pyruvate-oxime-2TMS	7.204	232.0, 247.0
Lactate-2TMS	6.198	219.0, 191.0
Citrate-4TMS	12.958	363.0, 347.0
Isocitrate-4TMS	12.951	465.0, 375.0
2-Ketoglutarate-meto-2TMS	11.212	198.1, 288.1
Succinate-2TMS	8.934	247.0, 172.0
Malate-3TMS	10.427	233.0, 189.0
Citrate-d $_4$-4TMS	12.936	367.2, 350.1
2-Isopropylmalate-3TMS	11.163	349.1, 377.1
